# Systemic semaglutide provides a mild vasoprotective and antineuroinflammatory effect in a rat model of ocular hypertensive glaucoma

**DOI:** 10.1186/s13041-025-01224-8

**Published:** 2025-07-01

**Authors:** Zaynab A. Mouhammad, Anne Rombaut, Mariana Yolotzin García Bermúdez, Rupali Vohra, James R. Tribble, Pete A. Williams, Miriam Kolko

**Affiliations:** 1 Department of Drug Design and Pharmacology, University of Copenhagen, Universitetsparken 2, Copenhagen, 2100 CPH Denmark; 2 Department of Clinical Neuroscience, Division of Eye and Vision, St. Erik Eye Hospital, Karolinska Institutet, Stockholm, Sweden; 3 Department of Ophthalmology, Copenhagen University Hospital, Rigshospitalet, Glostrup, Denmark; 4 Your Eye Doctors, Vestre Stationsvej 12, Rungsted, Denmark

**Keywords:** Astrocytes, Glaucoma, GLP-1 receptor agonists, Neuroprotection, Neurodegeneration, Neuroinflammation, Retina, Retinal ganglion cell, Semaglutide

## Abstract

Glaucoma is a neurodegenerative disease affecting retinal ganglion cells (RGCs), with a multifactorial genesis that includes inflammation and vascular dysfunction. Emerging evidence suggests that glucagon-like peptide 1 receptor agonist (GLP-1RAs) may serve as promising neuroprotective agents in glaucoma. In this study, we investigated the neuroprotective potential of the GLP-1RA semaglutide (SEM) in a rat model of ocular hypertension (OHT) induced by paramagnetic bead injections in Brown Norwegian rats. Rats were divided into four cohorts, two normotensive (NT) cohorts, and two OHT cohorts, treated with either SEM or saline (HBSS), which served as control. Systemic SEM or HBSS administration was initiated simultaneously with OHT induction. We observed that SEM administration seemed to delay the increase in intraocular pressure (IOP) associated with OHT. Although SEM administration did not improve RGC survival, it significantly improved astrocytic fractal dimension value and lacunarity. In conclusion, our findings suggest that GLP-1RAs may exert neuroprotective effects by delaying IOP elevation and preventing OHT-induced reactive astrocyte and vascular remodeling. These findings highlight the potential of GLP-1RAs for retinal neuroprotection, but further studies are needed to elucidate their applicability in glaucoma.

**Supplementary Information:**

The online version contains supplementary material available at 10.1186/s13041-025-01224-8.

Glucagon-Like Peptide 1 Receptor Agonists (GLP-1RAs), originally developed for diabetes, are currently being investigated for their neuroprotective potential in neurodegenerative conditions, including glaucoma [1]. Glaucoma is the leading cause of irreversible blindness worldwide and is characterized by the progressive loss of retinal ganglion cells (RGCs) and their axons accompanied by visual field defects [2]. Additional neurodegenerative components of glaucoma include vascular dysfunction, metabolic changes, and neuroinflammation, which are recapitulated in a variety of animal models of glaucoma [3, 4]. Both surgical and medical anti-glaucomatous interventions currently focus on reducing the only evidence-based modifiable risk factor, the intraocular pressure (IOP). However, up to 42% of treated patients are still expected to experience monocular blindness [5]. Recent epidemiological studies by our group and others indicate that the use of GLP-1RAs in diabetic patients may reduce the risk of glaucoma [6–8]. Previous findings have indicated neuroprotective effects of GLP-1RAs in models of retinal degeneration. These effects include reduced inflammation [9, 10], improved mitochondrial biogenesis and function [11, 12], preservation of the blood-retinal barrier [13, 14], and increased retinal vascularization [15]. These findings support the hypothesis that GLP-1RAs may be appropriate neuroprotective candidates for neuroprotection in glaucoma. If GLP-1RAs demonstrate to be effective in models of glaucoma, they can potentially become effective agents to supplement current treatment strategies, as a much needed neuroprotective agent. In this study, we aim to assess the neuroprotective effects of the GLP-1RA semaglutide (SEM) following systemic administration. While other GLP-1RAs have shown to cross the blood-brain barrier [16], SEM has been found to interact with the brain primarily through the circumventricular organs (CVO) and brain regions adjacent to the CVO [17, 18]. Thus, we expect SEM to exert its effects systemically, rather than by crossing the blood-retinal barrier (BRB).

To assess the effects of SEM, we used an inducible rat model of glaucoma where paramagnetic beads were injected into the anterior chambers (intracameral) of the eye to block the drainage of aqueous humor and elevate the IOP [19, 20]. Seventeen Brown Norway rats (Rattus norvegicus, with a starting weight of ~ 250–320 g, 12–16 weeks) received bilateral injections according to a previously described protocol [19, 20]. IOP was measured prior to injection (day 0) and at 2–3 day intervals for a period of 14 days using a Tonolab rebound tonometer. Six rats did not receive any intracameral injections and served as normotensive controls. At Day 0, and twice a week for 14 days, rats were treated subcutaneously with either SEM (5 mg/kg) or the equivalent volume of saline only (HBSS). After 2 weeks, rats were euthanized, eyes enucleated, immediately fixed for 30 min in 4% PFA, then in 0.4% PFA, and flat mounted. Retinas were immunofluorescently labeled as previously described [19, 20]. *Z*-stack confocal images were acquired using an Axioscan 7 at 20x magnification. Six regions of the central retina (1000 μm from the optic nerve head) were imaged and then cropped to 150 × 150 µm^2^, for RGC and monocyte quantifications, or 300 × 300 µm^2^, for microglia quantifications. Monocytes were identified as Isolectin B4 + cells lacking projections, while microglia were identified as mononuclear cells with projections. Cell quantifications were performed using the manual cell counter plugin for ImageJ (expressed as average of the 6 images). A fractal dimension analysis of astrocytes was conducted on GFAP-stained images acquired from areas distant from retinal blood vessels. The images were subjected to fixed thresholding and analysed using the Fractal Box Count plug-in on ImageJ. For the vascular analysis, superficial vascular plexus vessels were manually reconstructed on Adobe Illustrator, masking the optic nerve head with a round circle (120 × 120 pixels). Junction density, lacunarity and total vessel length were analysed using the AngioTool software (vessel diameter: 4–10 and intensity: 15–255). The normality of experimental results was tested using the Shapiro-Wilk test, with each eye counting as one sample (n). Variance homogeneity was evaluated using Bartlett’s test. Comparison of means was performed using one-way ANOVA or Welchs ANOVA followed by posthoc multiple comparison test (Dunnett’s or Tukey’s based on comparison to only NT HBSS, serving as control, or across the groups). Based on Grubb’s test, data points with p-values below 0.05 were considered significant outliers and excluded from the presented results. Finally, the relative delta differences were calculated when comparing OHT HBSS and OHT SEM to NT HBSS and NT SEM.

Rats treated with SEM (NT SEM and OHT SEM) demonstrated a persistent weight loss throughout the two weeks of treatment (Fig. 1A), indicating that the administered SEM exerted a systemic effect. The average IOP of NT SEM (e.g., day 14: 19.97 mmHg) remained non-significantly different from the IOP of NT HBSS (e.g., day 14: 20.3 mmHg, ∆ = 1.8%, *p* > 0.05) throughout the two-week treatment (Fig. 1B). On day 3, the IOP of OHT HBSS (34.4 mmHg) was significantly higher from NT HBSS (16.9 mmHg, ∆ = 50.8%, *p* < 0.01), while OHT SEM (24.0 mmHg) remained non-significantly different to NT HBSS (∆ = 29.7%, *p* > 0.05) on day 3 and throughout the first week post-injection. A significant increase in the average IOP of OHT SEM (34.2 mmHg) was first observed on day 9 compared to NT HBSS (18.1 mmHg, ∆ = 47.1%, *p* < 0.01). On day 14, second week post-injections, the IOP of OHT HBSS (32.2 mmHg) remained significantly higher than NT HBSS (20.3 mmHg, ∆ = 36.9%, *p* < 0.01). On day 14, the IOP of OHT SEM (28.2 mmHg) was also higher than NT HBSS (20.3 mmHg, ∆ = 27.9%, *p* > 0.1), but not as a variable. These findings demonstrate that intracameral microbead injections significantly increased the IOP, and that SEM seem to delay this IOP-increase.

**Fig. 1:**
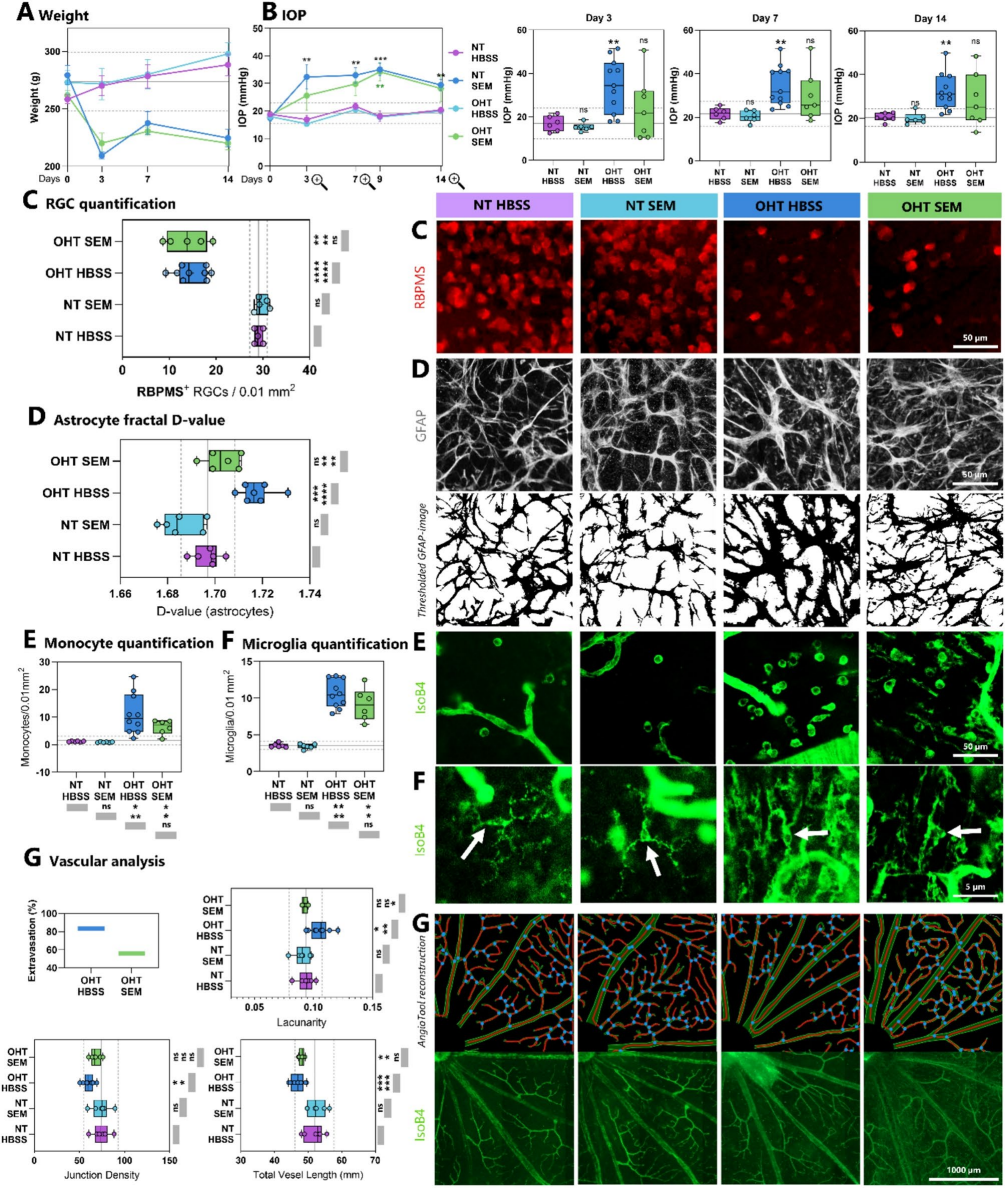
Systemic semaglutide does not prevent ocular hypertension induced RGC-loss, yet causes astrocytic remodeling, and some vasoprotection. Rats were administered either saline (HBSS) or semaglutide (5 mg/kg, SEM) subcutaneously simultaneous to the induction of ocular hypertension (OHT) using paramagnetic beads. The experimental groups were as follows: OHT treated with saline (OHT HBSS, rats = 6), normotensive controls (NT) treated with saline (NT HBSS, rats = 3), OHT treated with semaglutide (OHT SEM, rats = 5), and NT treated with semaglutide (NT SEM, rats = 3). **(A)** Rats treated with SEM, NT SEM and OHT SEM, exhibited a sustained weight loss throughout the two weeks. **(B)** Intraocular pressure (IOP) increased more slowly in SEM-treated OHT rats, compared to untreated OHT rats. **(C)** Retinal ganglion cell (RGC) quantification, based on RBPMS staining, showed no significant difference between SEM treated and untreated rats with OHT. **(D)** Astrocytic fractal dimension, assessed through GFAP staining, was significantly lower in SEM-treated OHT rats compared to OHT rats treated with saline. **(E)** No significant changes were observed in microglial density or **(F)** monocyte density between OHT groups. Compared to NT rats, both monocyte and microglial densities were significantly increased in both OHT groups, with microglia exhibiting a morphological shift from a ramified to an amoeboid form as indicated by the white arrows. **(G)** Flat-mounted retinas labeled with Isolectin B4 were used to image blood vessel morphology. Analysis revealed significantly increased total vessel length in both SEM-treated and saline-treated OHT rats, indicating OHT-induced vascular remodeling. However, SEM treatment appeared to decrease lacunarity and percentage of images with visible extravasation in rats with OHT. **RBPMS**: RNA-binding protein with multiple splicing, **GFAP**: Glial fibrillary acidic protein, **IsoB4**: Isolectin IB4, **NT**: Normotension, **OHT**: ocular hypertension, **SEM**: semaglutide, **HBSS**: Hank’s Balanced Salt Solution, **IOP**: Intraocular pressure. Scale bars = 50 μm in C, D and E, 5 μm in F, and 1000 μm in G. *P < 0.05, **P < 0.01, ***P < 0.001, ****P < 0.0001. NS, not significant (*P* > 0.05)

There were significantly fewer RGCs in both OHT HBSS (14.8 cells/0.01 mm^2^) and OHT SEM (13.8 cells/0.01 mm^2^) compared to NT HBSS (29.1 cells/0.01 mm^2^, ∆(OHT HBSS) = 49.1%, *p* < 0.0001, and ∆(OHT SEM) = 51.4%, *p* < 0.01) and NT SEM (∆(OHT HBSS) = 50.2%, *p* < 0.0001, and ∆(OHT SEM) = 53.7%, *p* < 0.01) (Fig. 1C). No significant differences in RGC density was found between OHT HBSS and OHT SEM (∆ = 7%, *p* = 0.997). These findings indicate that the OHT model significantly reduced RGC viability, and that two weeks of systemic SEM treatment did not rescue OHT-induced RGC degeneration.

While assessing the inflammatory parameters, OHT HBSS (1.717) had a significantly higher fractal dimension value compared to NT HBSS (1.697, ∆ = 1.2%, *p* < 0.001) and NT SEM (1.686, ∆ = 1.8%, *p* < 0.0001). OHT SEM (1.703) exhibited a significantly lower astrocyte fractal dimension value compared to OHT HBSS (1. 717, ∆ = 0.8%, *p* < 0.01) (Fig. 1D), and was not significantly different from NT HBSS (1.697, ∆ = 0.35%, *p* > 0.05) nor NT SEM (1.686, ∆ = 1%, *p* > 0.05). This demonstrates that SEM may hamper OHT-induced astrocyte remodeling.

Monocyte infiltration was significantly increased in both OHT-groups (OHT HBSS: 11.1 cells/0.01 mm^2^, OHT SEM: 6.3 cells/0.01 mm^2^) compared to NT HBSS (1.2 cells/0.01 mm^2^, ∆(OHT HBSS) = 89.6%, and ∆(OHT SEM) = 81.9%, *p* < 0.05) and to NT SEM (0.88 cells/0.01 mm^2^, ∆(OHT HBSS) = 92.1%, *p* < 0.01, and ∆(OHT SEM) = 86.1%, *p* < 0.05) (Fig. 1E). On average, OHT SEM (6.3 cells/0.01mm^2^) exhibited a lower monocyte density compared to OHT HBSS (11.1 cells/0.01mm^2^), but not as a variable (∆ = 42.95%, *p* > 0.05). These results indicate that OHT induces monocyte infiltration, and that SEM may slightly reduce this infiltration.

Microglial density was similarly significantly increased in both OHT-groups (OHT HBSS: 10.6 cells/0.01 mm^2^, OHT SEM: 9.1 cells/0.01 mm^2^) compared to NT HBSS (∆(OHT HBSS) = 66.5%, *p* < 0.0001, ∆(OHT SEM) = 61.2%, *p* < 0.01) and NT SEM (∆(OHT HBSS) = 67.5%, *p* < 0.0001, ∆(OHT SEM) = 62.3%, *p* < 0.01) (Fig. 1F). OHT SEM (9.1 cells/0.01 mm^2^) had a lower microglial density compared to OHT HBSS (10.6 cells/0.01 mm^2^, ∆ = 13.7%, *p* > 0.05), but not as a variable. These results indicate that OHT induces microglial infiltration, and that SEM may slightly reduce this infiltration.

83% of the retinas from OHT HBSS exhibited retinal extravasation and OHT SEM 55.6% (Fig. 1G). The superficial vascular plexus demonstrated a significantly lower lacunarity in OHT SEM (0.09) compared to OHT HBSS (0.11, ∆ = 12.1%, *p* < 0.05), but no significant difference to NT HBSS (0.094, ∆ = 1%, *p* > 0.05) and NT SEM (0.09, ∆ = 2.3%, *p* > 0.05). A lower lacunarity in SEM–treated OHT rats indicates that SEM may promote the preservation of a homogeneous and organized retinal vasculature, with fewer large gaps and irregularities in vessel distribution.

Junction density was lower in OHT HBSS (60.3 junctions/mm^2^) compared to OHT SEM (67.8 junctions/mm^2^), but not as a variable (∆ = 11%, *p* > 0.05). Although the higher junction density in OHT SEM was not significantly different from that in OHT HBSS, it may suggest that SEM helps reduce vessel loss, capillary dropout and retinal ischemia.

Compared to NT HBSS (51.9 mm) and NT SEM (52.5 mm), there was a significantly lower total vessel length in both the OHT HBSS (46.8 mm, ∆ = 10% and ∆ = 11%, *p* < 0.001) and OHT SEM (48.1 mm, ∆ = 7.2% and ∆ = 8.3%, *p* < 0.05), indicating vascular remodeling in both OHT-groups. No significant difference in total vessel length was found between OHT HBSS and OHT SEM (∆ = 2.9%, *p* > 0.05).

We hypothesized that systemic SEM treatment would exert protective effects on the retina by preventing RGC loss, astrocyte remodeling, and vascular changes. Although we did not expect SEM to cross the BRB, we anticipated that its retinal effects likely arise from indirect signaling via the CVOs or peripheral pathways, such as the increased vascular permeability associated with this OHT model [20]. Overall, our data suggest that systemic administration of SEM, initiated on the same day as OHT was induced, does not prevent the OHT-associated RGC-loss, but delays the magnitude of IOP increase, delays or partially prevents astrocyte remodeling, and provides mild vasoprotection. The delayed IOP increase corresponds to findings in previous studies on patients [21] and experimentally induced glaucoma [10]. The decrease in astrocyte remodeling in OHT eyes treated with SEM is consistent with a previous study of experimental glaucoma where systemic administration of the GLP-1RA NLY01 decreased reactive astrocyte transformation [9]. The vasoprotective effect of SEM also corresponds to a previous study where SEM decreased vascular leakage in a mouse model of diabetic retinopathy [14]. Thus, these data suggest that systemic SEM may provide mild multifactorial protection in glaucoma. To fully assess this, future studies should explore additional time points post IOP increase in various glaucoma and RGC injury models as well as assess dosing parameters to increase efficacy (e.g. whether treatment prior to insults would be relevant, route of administration, and concentration). Furthermore, to significantly strengthen the mechanistic understanding of how systemic SEM may reach the retina, further in vivo pharmacokinetic or in vitro permeability studies are required.

In conclusion, our results support that systemically administered SEM may cause a systemic effect leading to a delayed increase in IOP, prevention of IOP-induced astrocyte remodeling and some retinal vasoprotection. However, further studies are needed to fully understand the preventative effects of GLP-1RAs in glaucoma development and progression.

## Electronic supplementary material

Below is the link to the electronic supplementary material.

Supplementary Material 1

## Data Availability

The datasets used and/or analyzed during the current study are available from the corresponding author on reasonable request.
