# Supplementary material for: Systemic semaglutide provides a mild vasoprotective and antineuroinflammatory effect in a rat model of ocular hypertensive glaucoma
Source: Mol Brain. 2025 Jul 1;18:54. doi: 10.1186/s13041-025-01224-8 (PMC12211916; doi:10.1186/s13041-025-01224-8)
Supplement: Supplementary file 1 — Supplementary Material 1 [file 13041_2025_1224_MOESM1_ESM.docx]

**Rat strain and husbandry**

17 adult male Brown Norway rats (12–16 weeks old, weighing ̴ 250–375 g; SCANBUR, Karlslunde, Denmark) were kept under a 12-hour light/dark cycle and provided with ad libitum access to food and water. All experimental procedures followed the ARVO Statement for the Use of Animals in Ophthalmic and Vision Research. Individual study protocols were approved by the Stockholm Committee for Ethical Animal Research (10389-2018). Rats were randomly assigned to four groups: two normotensive (NT) groups and two ocular hypertensive (OHT) groups. The rats received either semaglutide treatment (NT SEM and OHT SEM) or Hanks’ Balanced Salt Solution (HBSS) (OHT HBSS and OHT HBSS) twice a week for 2 weeks. The total volume of the injection ranged from 1 to 1.65 mL, depending on the rat’s body weight. The injections were administered subcutaneously into both the left and right flank, with the total volume equally split between the two sites. The semaglutide dose was 5 mg/kg body weight (BW), based on a previous study that evaluated another GLP-1 receptor agonist, NLY01 (PMID: 33147455).

**Induction of ocular hypertension**

Ocular hypertension (OHT) was induced bilaterally (n = 6 rats for OHT HBSS, n = 5 for OHT SEM). OHT was induced using a magnetic microbead injection model, following previously established protocols to ensure reproducibility and consistency across experiments (PMID: 33510960, 38010699, 33510961). Microbeads (4.5-µm diameter; Dynabeads M-450 Epoxy; Thermo Fisher Scientific, Waltham, MA) were prepared for injection by washing with 1× Hanks’ Balanced Salt Solution (HBSS, no calcium, no magnesium, no phenol red; Thermo Fisher Scientific) and resuspended in a 0.25× volume of HBSS (∼1.6 × 10^6^ beads/µL). Rats were anesthetized with an intraperitoneal injection of ketamine (37.5 mg/kg) and medetomidine hydrochloride (1.25 mg/kg). Using a NanoFil syringe (World Precision Instruments, Sarasota, FL) with a 33-gauge tribeveled needle, 6 to 8 µL of bead solution was injected into the anterior chamber of the eye using a tunnel incision through the cornea. Beads were distributed to block the iridocorneal angle using a handheld magnet (cylindrical with a 4 mm diameter). Following surgery, on the same day, rats received treatment with either semaglutide or HBSS.

IOP was measured using a TonoLab rebound tonometer (Icare, Vantaa, Finland) in awake and unrestrained rats, as previously described (33510960, 38010699, 33510961). Rats were habituated to the tonometry procedure the week before surgery through familiarization with extended handling and tonometry training. Baseline IOP was recorded on the day of surgery (preoperatively, awake and unrestrained; day 0) and recorded every 2 to 4 days afterward until the endpoint (postoperative day 14), with IOP recordings always being taken between 9 AM and 10 AM to avoid the effects of circadian rhythm on IOP (2–3 hours from lights on). IOP was taken as the average of five tonometer readings. NT HBSS (n = 3 rats) and NT SEM (n = 3 rats) rats followed the same 14-day time course, just without surgery.

**Immunofluorescence**

Immunofluorescent labeling was performed following previously established standardized protocols (33510960, 38010699, 33510961). Rats were heavily anesthetized at day 14 by intraperitoneal injection of pentobarbital (75 mg/kg) and euthanized by cervical dislocation. Eyes were immediately enucleated and immersed in 3.7% paraformaldehyde (PFA) in 1× phosphate-buffered saline (PBS). Post-fixation, retinas were isolated as retinal flatmounts after careful removal of the cornea, lens, sclera, choroidea, optic nerve and corpus vitreum. Tissue was isolated using a hydrophobic barrier pen (Avantor, Radnor, PA), permeabilized in 0.5% Triton X-100 in 1× PBS for 1 hour, and blocked in 5% bovine serum albumin in 1× PBS for 1 hour; primary antibody was applied for at least 20 hours at 4°C. Tissue was then washed for 5 × 5 minutes in 1× PBS, and secondary antibody applied (1:500 in 1× PBS) for 4 hours at room temperature, followed by washing for 5 × 5 minutes in 1× PBS. After washing for 5 minutes in 1× PBS, the tissue was dried slightly, and mounted using Fluoromount-G and glass coverslips (Thermo Fisher Scientific). Glass coverslips were sealed using nail varnish. The antibodies and stains used for immunofluorescence labeling are detailed in Table I.

**Image acquisition and analysis**

Images were acquired using a ZEISS Axioscan 7 (20× magnification, scaling: 0.172 μm/pixel, Z-stacks: 15 slices with a thickness of ̴42 μm, and 3 channels: 112 HE, 43 HE and 38 HE), assigned an automated number and the identities of the groups concealed. All images were coded and analyzed under blinded conditions to prevent knowledge of exposure during quantifications and vessel analyses. After image acquisition, all acquired images were used to crop 6 images of fixed sizes (150 µm or 300 µm) around the central retina, within 0-1000 µm from the optic nerve head. All channels were cropped with Z-stacks to ensure optimal analysis of microglia, monocytes, retinal ganglion cells (RGCs) and astrocytes. For astrocytes, images were cropped around retinal vessels to avoid high fractal dimension values caused by astrocytic foot processes. ZEISS ZEN 3.9 was used for the cropping.

RGCs, microglia and monocytes were manually quantified using FIJI Image J Cell counter plug in. RBPMS-positive cells were identified as indicative of RGCs. Isolectin B4+ Mononuclear cells with projections were identified as microglia, while mononuclear cells lacking projections were identified as monocytes. A fractal dimension analysis of astrocytes was conducted on GFAP-stained images acquired from areas around retinal blood vessels. GFAP-stained images were subjected to fixed thresholding and analysed using the Fractal Box Count plug-in on FIJI Image J.

For retinal blood vessel analysis, manual thresholding of the Isolectin B4/blood vessel channel was performed on Inkscape. To ensure consistency, retinas were first cropped to 4,000 µm², covering more than 80% of the total retinal explant area. Blood vessel morphology was analyzed using AngioTool, which measured several parameters, including vessel coverage area, average vessel thickness, total blood vessel length, total vessel area, average vessel length, total vessel endpoints, total junctions, junction density, and mean lacunarity of the thresholded images. Lacunarity, a measure of space-filling within the image, indicates that higher values correspond to reduced blood vessel coverage. The AngioTool analysis was performed using the following parameters: blood vessel diameter (4–10 µm) and pixel intensity range (15–255).

**Statistical Analysis**

All statistical analyses was performed in Graphpad Prism 10, and pre-specified, including the choice of tests, significance thresholds, and criteria for data inclusion. Data were tested for normality with a Shapiro-Wilk test and then analyzed by one-way ANOVA followed by Tukey’s multiple comparison test. Unless otherwise stated, statistical significance levels correspond to: *P < 0.05, **P < 0.01, and ***P < 0.001, NS: non-significant (P > 0.05). For box plots, the center hinge represents the median with upper and lower hinges representing the first and third quartiles; whiskers represent 1.5 times the interquartile range.

| Table I. Primary and secondary antibodies | | | | |
| --- | --- | --- | --- | --- |
| Antibody | Target | Host | Dilution | Manufacturer (Catalog No.) |
| Isolectin GS-IB4 (IsoB4) | Poly-N-acetyllactosamine, found on microglia, endothelial cells, monocytes/macrophages | Lectin from Griffonia simplicifolia conjugated to biotin | 1:200 | Invitrogen (I21414) |
| RBPMS | RNA-binding protein, RGC specific in the retina | Rabbit | 1:250 | Novus Biologicals (NBP2-20112) |
| GFAP | Glial fibrillary acidic protein | Rabbit | 1:500 | Abcam (ab207165) |
| Streptavidin AF 488 conjugate | Biotin | Goat | 4 μg/ml | Invitrogen (S11223) |
| Goat-Anti Rabbit Alexa Fluor 568 | Rabbit primary antibody | Goat | 1:500 | Abcam (A11011) |
| Goat anti-Chicken AF 647 | Rabbit primary antibody | Goat | 1:500 | Abcam (ab150171) |
